# Investigating Age-Dependent Oxygenation and Blood Perfusion in a Mouse Model of Peripheral Artery Disease (PAD) Using Multispectral Optoacoustic Tomography (MSOT), Laser Speckle Contrast Imaging (LSCI) and Histology †

**DOI:** 10.3390/diagnostics16121783

**Published:** 2026-06-09

**Authors:** Bushra Afzal, Vy Tran, Na Nguyen, Savannah Qui-Tam Le, Tam Nguyen, Kytai T. Nguyen, Li Liu, Ralph P. Mason

**Affiliations:** 1 Prognostic Imaging Research Laboratory, Department of Radiology, University of Texas Southwestern Medical Center, 5323 Harry Hines Blvd, Dallas, TX 75390, USA; bushrafzal19@gmail.com; 2 Nanomedicine and Tissue Engineering Laboratory, Department of Bioengineering, University of Texas, 500 UTA Blvd, Arlington, TX 76010, USA; vy.tran@utsouthwestern.edu (V.T.); na.nguyen2@mavs.uta.edu (N.N.); sql8459@mavs.uta.edu (S.Q.-T.L.); tamphung.nguyen@uta.edu (T.N.); knguyen@uta.edu (K.T.N.)

**Keywords:** peripheral artery disease (PAD), multispectral optoacoustic tomography (MSOT), photoacoustic imaging, laser speckle contrast imaging (LSCI), histology, Masson’s trichrome, CD31, Ki67

## Abstract

**Background/Objectives:** Peripheral artery disease (PAD) is frequently asymptomatic, requiring non-invasive approaches for disease evaluation and therapy monitoring. This study demonstrates that multispectral optoacoustic tomography (MSOT) and laser speckle contrast imaging (LSCI) can non-invasively assess changes in tissue vascular oxygenation and perfusion, respectively, in a mouse hindlimb PAD model, enabling comparison of age-dependent vascular responses. **Methods:** PAD was induced by cauterization of the femoral artery in young (2 months) and old (18 months) mice, which were imaged using MSOT and LSCI at baseline (Day 0) and on Days 3, 7, and 14 post-surgery. Correlative histology including Hematoxylin and Eosin (H&E), Masson’s Trichrome for collagen, and immunofluorescence for CD31 and Ki-67 were performed. **Results:** Reduced tissue oxygenation was observed by MSOT in the ischemic limb shortly after surgery and faster recovery occurred in young compared to old mice. LSCI revealed time-dependent perfusion recovery in both groups, with consistently better recovery in young mice. Histological analyses confirmed ischemic damage and demonstrated enhanced angiogenesis and cellular proliferation in young muscle tissues. The observations were consistent for each methodology. **Conclusions:** These results indicate that both MSOT and LSCI serve as effective, non-invasive tools for longitudinal monitoring of muscle injury, capable of revealing age-dependent vascular responses without the need for exogenous contrast agents.

## 1. Introduction

Peripheral artery disease (PAD) is a common circulatory disease worldwide estimated to affect almost 15% of older people with somewhat higher incidence in women [1]. High blood pressure, high cholesterol, diabetes mellitus, chronic renal insufficiency, age, and smoking are major risk factors for PAD. Patients with hypercholesterolemia frequently seek assistance in controlling their lower limb vascular disease following an acute thrombotic or embolic episode. The 5-year cumulative incidence of clinical deterioration from asymptomatic PAD to intermittent claudication (IC) is 7%, and from IC to chronic limb-threatening ischemia (CLTI) is 21% [2] with a major amputation rate of 9% in patients with CLTI [3]. Endovascular revascularization and traditional surgical procedures are effective therapeutic options for many patients. However, due to severe comorbidities or the complex anatomy of the vascular occlusion, about 30% of patients are not eligible for surgical or interventional operations, making non-surgical treatment important [4,5]. An opportunity to detect PAD earlier and monitor therapeutic interventions could benefit clinical practice and patient outcomes.

Photoacoustic imaging (PAI) is an emerging technology that combines the high spatial resolution of ultrasound imaging with the molecular sensitivity of optical imaging [6,7]. PAI is based on pulsed optical stimulation of molecules, whereby rapid thermoelastic excitation generates acoustic waves that can be detected by ultrasound. Varying concentrations of chromophores, notably oxy- and deoxyhemoglobin (HbO_2_ and dHb, respectively), provide tissue contrast in images. The absorption spectra of oxy- and deoxyhemoglobin are unique, enabling direct visualization and quantification of hemoglobin concentration [Hb] and oxygen saturation (sO_2_), along with anatomical detail for imaging vascular structures [8,9,10].

PAI is ideally suited to examining tissue vascular development and impairment and has been widely applied in investigations of cancer and novel treatments [11,12,13]. Application to PAD is less common, although there are a few reports tracking limb oxygenation and vascular dysfunction in peripheral arteries in both pre-clinical and human studies [9,10,14,15,16,17,18]. PAI has a number of benefits over more traditional imaging techniques; specifically, it avoids the radioactivity required for tracers in nuclear medicine methods such as position emission tomography (PET) or single photon emission computed tomography (SPECT) or the external radiation beam for computed tomography (CT). Magnetic Resonance imaging (MRI) often uses gadolinium-based contrast agents, which are being restricted in the light of nephrogenic fibrogenic syndrome (NFS). Above all, PAI typically uses a hand-held transducer, which is much cheaper than PET, SPECT, CT or MRI and does not require the patient to lie within an instrument bore, which can be claustrophobic. Ultrasound uses a similar hand-held device and is still cheaper and simpler, but tissue contrast is limited to tissue boundaries, unless a microbubble contrast agent is applied. PAI uses wavelength selective excitation and detects the acoustic signals generated by transient molecular thermoelastic expansion, thus providing spatial resolution for optical imaging. Further, by exciting multiple wavelengths sequentially (multispectral optoacoustic tomography (MSOT)), the differential absorption of specific chromophores can be observed via spectral unmixing. Notable chromophores include oxy- and deoxyhemoglobin, fat, melanin, and water [19]. In some instruments ultrasound excitation/capture is also available providing traditional tissue contrast based on the ultrasound alone [20]. Two primary pre-clinical instruments are available, specifically, the iThera InVision and Fuji Vevo LAZR-X [13]. The LAZR-X system uses integrated ultrasound and optical excitation/detection based on a hand-held transducer, but there is signal attenuation with depth limiting investigations. Meanwhile, the InVision system immerses the subject (mouse) in a water bath, allowing toroidal excitation and detection, yielding a more uniform signal throughout a transaxial slice of the subject. The surrounding water maintains mouse temperature and physiology, but requires breathing through a snorkel and the subject diameter is limited to about 3.5 cm. The mouse must be immobilized, typically by anesthesia, but this allows a gas breathing challenge. There was also a commercial Endra Nexus system, which immersed mouse in water bath and provided 3D volumetric PAI, but required the whole mouse to be depilated. Various experimental systems have also been reported from specific research laboratories.

Laser speckle contrast imaging (LSCI) is also a non-invasive technique for assessing blood flow without the need for exogenous contrast agents [21]. It enables real-time, high-speed visualization of blood flow and perfusion making it ideal for continuous or dynamic monitoring during surgery or therapy. Being an optical only system, LSCI is especially effective for imaging superficial blood flow, allowing assessment of skin perfusion, wound healing, and microvascular dynamics.

This study investigated age-dependent changes in a mouse model of PAD. Female mice from two distinct age groups were analyzed to assess age-related differences in ischemic limb oxygenation and blood perfusion using MSOT and LSCI, respectively. Histological analyses were conducted to confirm structural and compositional alterations in healthy and ischemic gastrocnemius limb muscle after PAD induction.

## 2. Material and Methods

BALB/c female mice from two age groups (2 months and 18 months) were used to study a PAD model for comparing oxygen saturation and blood perfusion between young and old mice. Sixteen mice (8 young and 8 old) were used for oxygenation studies using MSOT, and 24 mice (8 young and 16 old) were used for LSCI to assess blood perfusion. Half the mice used for MSOT imaging were also used for histological analysis after completing imaging at the final time point (Day 14). Mice were housed in AAALAC accredited facilities, up to 5 per cage in a ventilated rack housing system with automatic water and food ad libitum. Temperature was maintained in the range 20 °C to 24 °C with relative humidity at 45 to 65% and a 12 h light/dark cycle.

### 2.1. Creation of Hindlimb Ischemia Model

All animal experiments were approved and performed in accordance with the UTSW and UTA Animal Care and Use Committee (IACUC) guidelines under protocols APN: 2021-103082 or A21.003. Female BALB/c mice (2 months and 18 months) were purchased from Inotiv, Inc. (West Lafayette, IN, USA). A mouse model of unilateral hindlimb ischemia was used with slight modification (cauterization in place of ligation), as previously described [22,23]. Mice were anesthetized and maintained with isoflurane (~2%). Carprofen (100 µL) and Buprenorphine SR (30 µL) were given just before the surgery. The left femoral artery and its branches were exposed and cauterized, and the skin was subsequently closed with absorbable polyamide nylon sutures 5-0 (Covetrus North America, Dublin, OH, USA). The right hindlimb served as a non-ischemic control. Mouse cages were placed on a heating pad after surgery to enhance recovery and maintain a normal temperature of 37 °C. Following surgery, Buprenorphine SR was administered up to total 10 doses. Separate cohorts of mice were used for LCSI and MSOT investigations, but the surgery was undertaken by a single skilled surgeon.

### 2.2. Multispectral Optoacoustic Tomography (MSOT)

To obtain optimal acoustic and optical contact, the legs were shaved and carefully depilated with Veet cream containing Aloe Vera (Reckitt, Nutley, NJ, USA). While VEET was applied before each imaging session, exposure was brief (<60 s) and it was removed with gentle water-cleansing, with no obvious skin irritation or inflammation dung the course of the study. Mice were anesthetized and given isoflurane (~2%) in air or oxygen via a nose cone (snorkel). MSOT (iThera InVision-256 (Munich, Germany)) was used for imaging. The mouse was placed in holder surrounded by thin plastic film and acoustic gel (Parker Aquasonic clear ultrasound gel, Fairfield, NJ, USA) was used to ensure effective optical and acoustic contact. The holder was placed in the imaging chamber, surrounded by water (34 °C), and following 10 min thermal equilibration, photoacoustic images and oxygen saturation maps were acquired using 7 wavelengths (680, 730, 760, 798, 874, 930, 972 nm). Each laser pulse required 100 ms and 5 shots were applied per wavelength, so that each sO_2_ map was acquired in about 3.5 s and 30 sequential images were acquired. The oxygen gas-breathing challenge was based on 5 min Air, 5 min Oxygen and 3 min return to Air. Mice (8 old and 8 young) were imaged on four occasions: Day 0 (pre-surgery), Day 3, Day 7 and Day 14 to evaluate surgical (ischemic) and control (non-ischemic) thighs, as well as spine muscle. Images were reconstructed, fluence corrected, and unmixed at preset 4.0 BP in vivo at a field of view of 25 mm (res: 75 µm) in ViewMSOT 4.0 software. Molecular concentrations achieved by MSOT are not strictly quantitative and thus values are sO_2_^MSOT^. Relative changes are much more reliable and thus changes in sO_2_ (ΔsO_2_) in response to an intervention, such as a gas breathing challenge, are often favored.

### 2.3. Laser Doppler Blood Perfusion Measurement

Blood perfusion of ischemic legs was measured using LSCI (PeriFlux 6000 Laser Doppler, Perimed, Las Vegas, NV, USA), as previously described [24]. Blood perfusion was monitored at several timepoints (Day 0, Day 1, Day 3, Day 7, and Day 14). The mice were anesthetized with 2% isoflurane inhalation and placed on a warm 37 °C plate before starting measurements, to minimize blood flow variations due to ambient temperature. The limb perfusion ratio was determined in 8 young and 16 old mice by calculating the ratio of the left (ischemic) leg to the right (healthy) leg. All mice were imaged at Day 0 (pre-surgery) and 1 day following surgery. Three young and 3 old mice were observed on each occasion together with additional subgroups.

### 2.4. Tissue Collection, Processing, and Embedding for Histology

At the designated endpoint, the first 4 young and 4 old mice from the MSOT study were selected for histological comparison. Mice were euthanized by cervical dislocation, and tissues were collected under sterile conditions dissecting gastrocnemius muscles from both the healthy (non-ischemic) and PAD (ischemic) hindlimbs of each mouse. Tissues were briefly rinsed in phosphate-buffered saline (PBS) to remove residual blood and minimize superficial dehydration. Tissues were placed in cassettes for subsequent fixation and histological processing. Tissue cassettes were submerged in appropriate solutions for processing. Following overnight fixation (10% neutral buffered formalin), cassettes were transferred to PBS and incubated for at least 30 min to remove residual fixative prior to dehydration. Tissue cassettes were dehydrated through a graded ethanol series (50–100%), cleared in xylene, and infiltrated with paraffin wax overnight. Tissues were further incubated in molten paraffin under vacuum for 1 h to enhance infiltration and then embedded in paraffin with vertical orientation to obtain transverse sections. Paraffin blocks were solidified on a cold plate, stored, and sectioned at 7 µm thickness. Sections were dried overnight, deparaffinized in xylene, and rehydrated through graded ethanol to deionized water. Slides were subsequently processed for H&E, Masson’s Trichrome, and immunofluorescence staining.

### 2.5. Hematoxylin and Eosin (H&E) Staining

Using a standard histological protocol [25], tissue sections were stained with H&E to visualize cellular and structural morphology. After staining, slides were mounted using Permount mounting medium and sealed with glass coverslips. Tissue images were acquired using the Hamamatsu NanoZoomer S60 (Hamamatsu Photonics K.K., Hamamatsu, Japan) digital slide scanner and analyzed using ImageJ (version 1.54r, 64-bit) software to quantify the percentage of adipose tissue coverage (5 regions of interest (ROIs)), allowing comparison between healthy control tissue and ischemic tissue (PAD).

### 2.6. Masson’s Trichrome Staining

Tissue sections were processed using reagents from American MasterTech Scientific, Inc. (Lodi, CA, USA) according to the manufacturer’s instructions. Slides were sequentially stained with working Weigert’s hematoxylin to visualize nuclei, followed by Biebrich Scarlet-Acid Fuchsin to stain cytoplasmic and muscle fibers. Differentiation of collagen was achieved using phosphotungstic/phosphomolybdic acid, after which collagen was stained with aniline blue. Following staining, sections were dehydrated through graded alcohols, cleared, and mounted using Permount mounting medium. Whole-slide images were acquired using the Hamamatsu NanoZoomer S60 scanner. The percentage area of collagen was quantified across the entire tissue section.

### 2.7. Method/Experimental

Tissues were stained for mature vasculature (CD31) and proliferation (Ki67). Tissue sections were deparaffinized, rehydrated, and subjected to antigen retrieval (AR) using either Tris/EDTA (pH 9.0) for CD31 or sodium citrate (pH 6.0) for Ki67. To block nonspecific binding, sections were incubated with 10% normal goat serum and 1% BSA in Tris-buffered saline (TBS) for 2 h at room temperature. Sections were incubated with anti-CD31 (PECAM-1) rabbit monoclonal primary antibody (Cell Signaling Technology, Danvers, MA, USA; cat. no. 77699) at 1:100 or 1:1000 dilution. For Ki67, rabbit monoclonal primary antibody (Cell Signaling Technology; cat. no. 12202) at 1:200 or 1:1000 dilution, respectively. Tissue sections were incubated with primary antibodies overnight in the refrigerator at 4 °C under a moist atmosphere. Sections were washed with TBS containing 0.025% Triton X-100, followed by TBS alone. After washing, sections were incubated with FITC-conjugated goat anti-rabbit IgG secondary antibody (Abcam, Cambridge, UK; cat. no. ab6716) for both primary antibodies for 1 h at room temperature. Nuclei were counterstained with 4’,6-diamidino-2-phenylindole (DAPI), with the dilution of 2 drops NucBlue per 1 mL TBS, before mounting with Fluoromount. Slides were scanned using a ZEISS Axio Scan.Z1 microscope (Carl Zeiss Microscopy GmbH, Jena, Germany). Quantitative analysis was determined by calculating the percentage of FITC-positive areas relative to the total tissue area in 5 ROIs.

### 2.8. Statistical Analysis

MSOT data were analyzed using GraphPad Prism version 10.3.0 (GraphPad Software, La Jolla, CA, USA), and histograms were generated in MATLAB R2023a. Oxygen saturation maps were determined and mean sO_2_ and mean ΔsO_2_ calculated. Mean ΔsO_2_ values were compared between groups, including spine, healthy, and PAD legs in young mice at various time points, as well as between healthy and PAD legs in young versus old mice. LSCI data were analyzed to compare the ratio of PAD to healthy legs between young and old mice, as well as between pre- and post-surgery time points. Fiji (ImageJ, version 1.54r, 64-bit) software was used for histological analysis. Two-way repeated-measures analysis of variance (ANOVA) was performed to assess the effects of age (young vs. old), exposure (air vs. oxygen), and limb condition (healthy vs. ischemic) across multiple time points, including interaction effects. A repeated measures analysis was applied for longitudinal comparisons in which the same animals were evaluated over time. Post hoc multiple comparisons were performed with appropriate corrections for multiple testing. All statistical analyses were performed using GraphPad Prism. Results are reported as standard error of the mean (SEM) and *p* < 0.05 was considered statistically significant based on Šídák’s multiple comparisons test.

## 3. Results

In both young and old mice, ischemic limbs appeared pale immediately after surgery due to low blood perfusion. Over the next few days, blood flow became more apparent, likely due to the dilation of collateral vessels in response to ischemia. One old mouse assessed by MSOT suffered necrosis of the injured leg leading to auto-amputation, but the other 15 mice survived intact for the whole study.

### 3.1. MSOT

**Figure 1:**
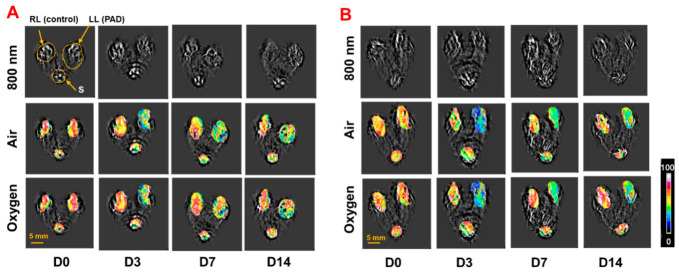
Vascular oxygenation observed using MSOT with respect to surgery in young and old mice. (Top row) Trans-axial single wavelength (800 nm) images. Gray scale with regions of interest at successive time points: Day 0 (baseline pre-surgery), and Days 3, 7 and 14 after surgery. (**A**) Young mouse (2 months), (**B**) old mouse (18 months). (Middle row) oxygen saturation (sO_2_^MSOT^) maps overlaid on gray scale while breathing air and, (bottom row) while breathing oxygen. RL: Right leg (control), LL: left leg (PAD), S: spine, as indicated by yellow arrows.

Multispectral optoacoustic tomography (MSOT) was successfully applied to examine trans-axial cross sections of cohorts of mice, specifically providing distributions of oxygen saturation (mean _S_O_2_^MSOT^) in the peripheral artery disease (PAD) model limb, the contralateral normal healthy limb, and spine of both young and old mice. Both thigh muscles and spine were readily identifiable, and the four main blood vessels of spine/tail were apparent (Figure 1A,B). At baseline, pre-surgery, the left and right legs appeared quite similar, and both leg muscles and spine showed an obvious significant increase in oxygen saturation upon breathing oxygen (*p* < 0.001). Following surgery, the PAD model leg appeared less oxygenated. The control tissues appeared quite similar over 14 days, while the injured leg responded much less to the oxygen gas breathing challenge (Figure 1A,B).

**Figure 2:**
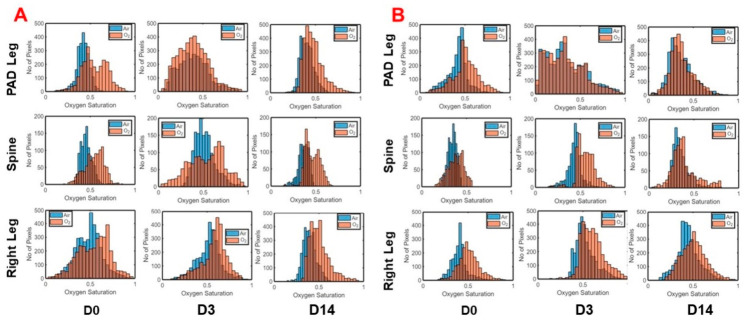
Vascular oxygenation determined by MSOT with respect to surgery. Pre-surgery (D0) and post-surgery (D3 and D14) histograms showing distribution of pixelwise oxygen–hemoglobin saturation (sO_2_^MSOT^) with respect to the oxygen gas challenge in (**A**) the young mouse from Figure 1; (**B**) old mouse from Figure 1.

**Figure 3:**
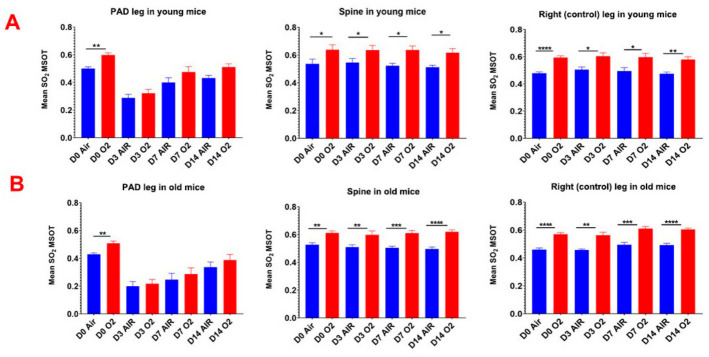
Vascular oxygenation with respect to surgery. Vascular oxygen saturation in response to an oxygen breathing challenge in right (control) leg, spine and left (PAD) leg over several days (D0, D3, D7 and D14) with respect to surgery in young and old mice: (**A**) Young mice (*n* = 8); (**B**) old mice (*n* = 8). Data presented as mean ± SEM, *p* ≤ 0.05 *, *p* ≤ 0.01 **, *p* ≤ 0.001 ***, *p* ≤ 0.0001 ****. Blue bars represent air breathing and Red bars represent O_2._

Both young and old mice showed substantial recovery over 14 days. Sequential mean sO_2_^MSOT^ values are shown for individual mice in Supplementary Figure S1. Histograms revealed the oxygen–hemoglobin saturation sO_2_^MSOT^ changes, in terms of frequency of pixels with respect to the oxygen gas challenges, with a significant left shift at Day 3 and considerable recovery at Day 14 in young and old mice (Figure 2A,B). Significant increases in mean _S_O_2_^MSOT^ were observed in both the spine muscle and healthy limb at all time points with respect to oxygen gas breathing challenge in both age groups (Figure 3A). In the PAD limb, a notable decrease in mean _S_O_2_^MSOT^ was observed on Day 3 post-surgery, with partial recovery by Day 14; however, values remained significantly different from baseline (Figure 3A). Mean Δ_S_O_2_^MSOT^ in PAD legs of old mice recovered more slowly than for young mice (Figure 3B).

**Figure 4:**
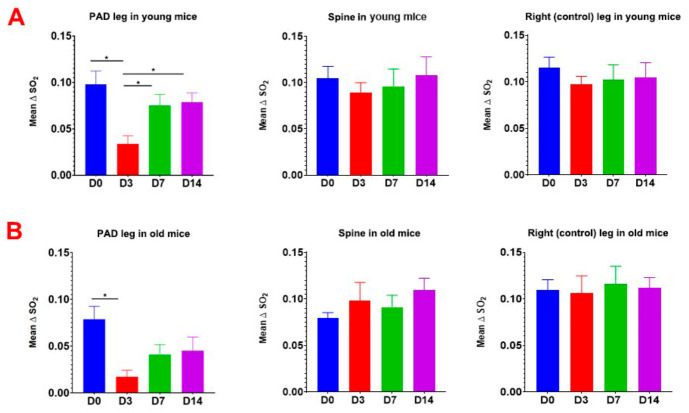
Vascular oxygenation recovery following surgery. Mean ∆sO_2_^MSOT^ for vascular oxygen saturation in response to an oxygen breathing challenge in right (control) leg, spine and PAD leg over several days (D0, D3, D7 and D14) with respect to surgery in young and old mice: (**A**) Young mice (*n* = 8); (**B**) old mice (*n* = 8). Data presented as mean ± SEM, *p* ≤ 0.05 *.

**Figure 5:**
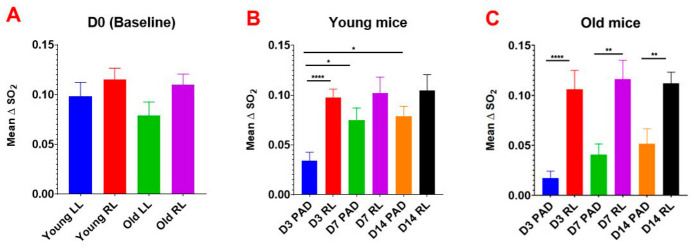
Vascular oxygenation response to oxygen gas breathing challenge comparing legs in young and old mice with respect to surgery. Mean ∆sO_2_^MSOT^ for vascular oxygen saturation in response to an oxygen breathing challenge in between right (control) leg and PAD leg over several days with respect to surgery. (**A**) Comparison of response in left and right legs at baseline in young (*n* = 8) and old (*n* = 8) mice prior to surgery; (**B**) comparison at specified time points in young mice (D0, D3, D7 and D14; *n* = 8); (**C**) comparison at specified time points in old mice (D0, D3, D7 and D14; *n* = 8 except D14 *n* = 7). Data presented as mean ± SEM, *p* ≤ 0.05 *, *p* ≤ 0.01 **, *p* ≤ 0.0001 ****.

The spine muscle maintained stable mean ΔsO_2_^MSOT^ across both age groups, with no significant differences. Significant differences in mean ΔsO_2_^MSOT^ were identified in the PAD limb of both young and old (*p* < 0.05) mice at Day 3 compared to Day 0. In young mice there was significant recovery by Day 7 (*p* < 0.05). On Day 14 the injured legs of both young and old mice were no longer significantly different from pre-surgery (Figure 4A,B). The control normal limb of both young and old mice maintained consistent oxygenation, with no significant changes (Figure 3 and Figure 4). Comparing young and old mice there were no significant differences in mean ΔsO_2_^MSOT^ for control leg or spine (Figure 4A,B). At baseline (pre-surgery) there was no significant difference between right and left legs in young or old mice (Figure 5A). At Day 3 following surgery significant differences were noted between injured and control legs for both young and old mice. By Day 7 PAD legs in young mice showed a significantly greater response than at Day 3 and they were no longer significantly different from control legs (Figure 5B). Old mice showed a significant difference in response between PAD and control legs throughout the study (Figure 5C). A strong dependency of sO_2_^MSOT^ (oxygen) on sO_2_^MSOT^ (air) was observed for the injured legs at each measurement (R^2^ > 0.94), while the ΔsO_2_^MSOT^ showed weaker correlation (R^2^ = 0.251) (Supplementary Figure S2). For the spine muscles and control legs only, weak correlations were observed.

### 3.2. LSCI

**Figure 6:**
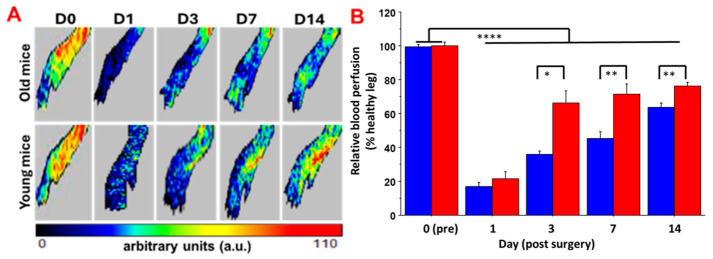
Laser speckle contrast imaging (LSCI) of blood perfusion. (**A**) LSCI images of injured paw showing blood perfusion over 2 weeks following surgery (D0, D1, D3, D7 and D14) in young (*n* = 8) and old mice (*n* = 16); (**B**) relative perfusion of PAD and control legs showing a trend of recovery in blood perfusion, which was significantly greater in young, as opposed to old mice. Blue: old mice; red: young mice. Data presented as mean ± SEM; *p* ≤ 0.05 *, *p* ≤ 0.01 **, *p* ≤ 0.0001 ****.

LSCI showed blood perfusion in the hindlimbs of young and old mice (Figure 6A). As expected, perfusion was similar in the control and experimental legs prior to surgery with a ratio of 1 at Day 0 (Figure 6B). The day after surgery, blood perfusion ratios dropped significantly to about 20% in both young and old mice (Figure 6B). Over time, blood perfusion improved differently in each group. By Day 3, young mice exhibited a significantly faster recovery, with blood perfusion reaching about 66%, whereas old mice showed slower recovery (about 36%; Figure 6B). Young mice continued to recover steadily, with about 76% blood perfusion by Day 14. In contrast, the old mice showed a slower recovery reaching only about 65% by Day 14, and no further improvement was observed after Day 14 up to Day 30. Not all mice were imaged on each occasion, but sequential perfusion measurements were acquired for three old and three young mice (Supplementary Figure S3), which indicates highly consistent behavior. The consequences of ischemia were more severe in the older mice, with increased rates of necrosis of the entire paw, resulting in limb auto amputation in addition to reduced blood perfusion, while young mice primarily developed toe and skin necrosis.

### 3.3. Hematoxylin and Eosin (H&E) and Masson’s Trichrome Staining

**Figure 7:**
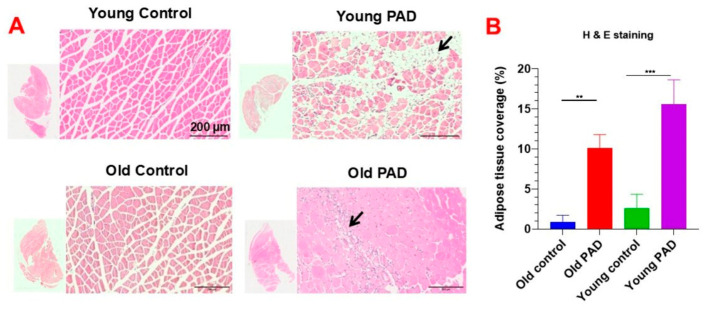
Hematoxylin and Eosin (H&E) stained mouse muscle tissues comparing injured and control conditions in young (*n* = 8) and old mice (*n* = 8). (**A**) Representative whole slice and ROI images of H&E staining highlights cytoplasm (pink), nuclei (dark purple/black), scale bar 200 µm. Black arrow indicates adipose area; (**B**) quantification of adipose tissue as a percentage of total tissue area. Values are mean ± SEM; *p* ≤ 0.01 **, *p* ≤ 0.001 ***.

Staining revealed distinct differences in tissue composition between mice with injured (PAD model) and healthy controls. In both young and old healthy mice, skeletal muscle fibers appeared densely packed and uniformly stained with Eosin, with nuclei positioned peripherally characteristic of mature, structurally intact muscle (Figure 7A). In contrast, injured gastrocnemius muscles exhibited disrupted fiber architecture, including centrally located nuclei, indicative of ongoing muscle regeneration. Additionally, injured tissues showed signs of inflammation, such as increased cell density and clustering of nuclei, reflecting the infiltration of immune cells. This inflammatory response is commonly associated with PAD pathology and contributes to further tissue damage and fibrotic remodeling (Figure 7A). Adipocytes were identified as large, round to oval cells with clear cytoplasmic spaces due to lipid extraction during processing and peripherally displaced nuclei. These cells typically appeared in clusters or lobules separated by thin connective tissue septa, highlighting adipose infiltration as a pathological feature in PAD muscle (Figure 7B). Injured legs exhibited a markedly higher proportion of adipose tissue compared to healthy controls, in both old and young mice (*p* < 0.005) (Figure 7B).

**Figure 8:**
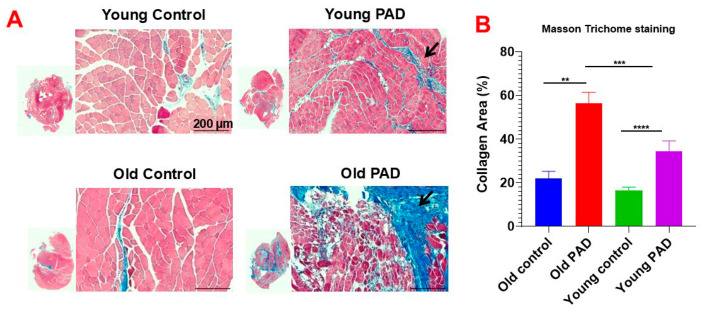
Masson’s Trichrome staining of mouse gastrocnemius muscle tissue comparing PAD and control groups in between young (*n* = 8) and old mice (*n* = 8); (**A**) representative whole slice and ROI images of Masson’s Trichrome staining differentiates muscle fibers (red), collagen fibers (blue), and cell nuclei (dark blue/black). Scale bar 200 µm, black arrow indicates collagen region; (**B**) quantification of collagen tissue as a percentage of total tissue area. Values are mean ± SEM; *p* ≤ 0.01 **, *p* ≤ 0.001 ***, *p* ≤ 0.0001 ****.

Masson’s Trichrome staining revealed collagen as blue regions, while viable muscle fibers were stained red (Figure 8A). In control tissues, nuclei were stained dark blue, whereas in ischemic PAD tissues, nuclei appeared darker. Compared to healthy controls, injured muscle displayed markedly increased blue-stained collagen, indicating a higher degree of fibrosis and scar tissue formation (Figure 8B). In old mice, there was a significantly greater increase in area of collagen than in young mice (*p* = 0.002). Injured tissue showed statistically greater fibrosis with respect to controls and old injured tissue showed more than young (*p* < 0.0001) (Figure 8B).

#### Immunofluorescence Staining

**Figure 9:**
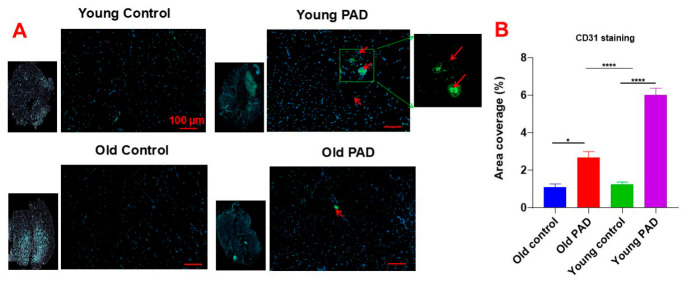
Immunofluorescence analysis of CD31 expression in mouse muscle tissue comparing peripheral arterial disease (PAD) and control conditions in young (*n* = 4) and old mice (*n* = 4). (**A**) Representative whole slice and ROI fluorescence images showing CD31 expression. Scale bar 100 µm, red arrows indicate CD31; (**B**) quantification of CD31-positive area as a percentage of total tissue area. Values are mean ± SEM; *p* ≤ 0.05 *, *p* ≤ 0.0001 ****.

**Figure 10:**
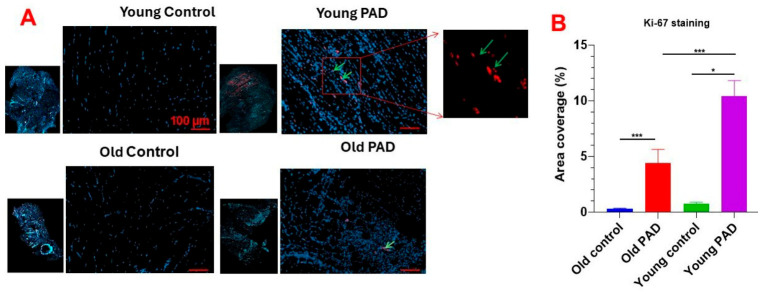
Immunofluorescence analysis of Ki67 expression in mouse muscle tissue comparing PAD and control conditions in young (*n* = 4) and old mice (*n* = 4). (**A**) Representative fluorescence images showing Ki67 expression. Scale bar 100 µm. Green arrows indicates Ki-67 signal. (**B**) Quantification of Ki-67-positive area as a percentage of total tissue area. Values are mean ± SEM; *p* ≤ 0.05 *, *p* ≤ 0.001 ***.

CD31-positive signals exhibited punctate and linear patterns along microvessels, reflecting the organization of endothelial cells and potential endothelial activation associated with inflammatory processes, with nuclei counterstained using DAPI (blue) (Figure 9A). Both young and old healthy muscles showed similar CD31 expression, but they were significantly higher in injured tissues, particularly in the young healthy controls. Significantly higher vascular density was observed in young PAD mice compared with old (*p* < 0.0001) (Figure 9A,B). Ki-67 staining in young and old mice revealed distinct expression patterns in injured tissues and nuclei stained with DAPI (blue) (Figure 10A). Ki67 staining indicated greater proliferative activity in PAD tissues compared to controls, with young PAD mice exhibiting higher expression than older PAD mice (*p* < 0.001)) (Figure 10B).

## 4. Discussion

We successfully evaluated a mouse model of peripheral artery disease based on unilateral cauterization of the femoral artery and imaging over a period of two weeks. Photoacoustic imaging revealed significant severe hypoxia in the injured hindlimb three days after surgery, which spontaneously recovered over the next 11 days. Meanwhile, the contralateral control leg and spine muscles each showed highly stable oxygenation over the four measurements over two weeks. There were subtle differences between young and old mice. Within seven days, the injured legs of young mice had recovered significantly compared with Day 3 and the response to an oxygen gas breathing challenge was similar to control legs, whereas it remained significantly depressed over the 14 days in the old mice. The increase in oxygenation was also significantly greater in young versus old injured legs at Day 7. LSCI showed similar vascular shutdown and recovery with young mice showing significantly faster and greater recovery.

MSOT is ideally suited to observing and measuring spatial distribution of vascular oxygenation, noninvasively, without the need for an exogenous reporter molecule [18]. However, we are aware of only three previous applications to hindlimb studies in mice [14,26,27]. Kirkham et al. examined vascular oxygenation in the skeletal muscles of normal young and old athymic nude mouse hindlimbs by PAI [26]. They used a Vevo LAZR 2100 system with just two wavelengths to estimate hemoglobin–oxygen saturation. They imaged along the long axis of the calf muscle and found significantly smaller perfused area in young mice, as compared with old mice. The fractional area of oxygenation was 60.6% vs. 6.0% [26]. However, the sO_2_ of the blood was very similar for both groups (50.0 ± 2.0% vs. 58.9 ± 3.1%). In the transaxial orientation, we saw no obvious difference in the perfused area for young vs. old mice. We saw similar sO_2_^MSOT^ in young and old mice pre-surgery while breathing air (50 ± 1% vs. 43 ± 1%, respectively, Figure 3) matching the previous report. A second study, from the same laboratory, examined acute response to cuff-induced ischemia and release. As a group, their mice had similar baseline sO_2_ (~48%), which decreased to about 33% within 3 min of occlusion and returned almost instantly upon release, with a transient overshoot to 75%, indicative of reactive hyperemia [14]. Individual mice showed a greater range of oxygenation. As opposed to acute ischemia, we examined chronic ischemia induced by cauterizing the femoral artery. At 3 days post-surgery, we observed quite similar oxygenation to the cuff occlusion (mean sO_2_ = 20 to 30%, Figure 3). However, we primarily examined change in sO_2_ (ΔsO_2_) in response to an oxygen gas breathing challenge, since this has been reported to be more closely aligned with hypoxia in tumors [28]. Hedhli et al. examined acute and chronic changes in vasculature following femoral artery ligation with multimodality imaging including PAI and LSCI [27]. Vascular oxygenation was assessed in the ischemic leg of male black C57BL/6 at various time points after ligation (20 min, 30 min, 40 min, 50 min, 60 min, 1, 2 and 7 days). They used a dual wavelength approach with an Endra system to measure the ratio of HbR (deoxyhemoglobin) to HbO_2_ and observed progressive hypoxiation over 20 min. By Day 2, they already observed vascular reoxygenation, which largely returned to baseline after 7 days. They also noted a continual increase in CD31 in the muscles over 2 weeks. We observed vascular recovery up to Day 14 (Figure 3 and Figure 4).

There have been limited studies using PAI in human calf muscle after inducing ischemia with a cuff as a model of PAD. Karlas et al. reported that PAD patients had significantly lower levels of vascular oxygenation compared to healthy volunteers, but following revascularization surgery oxygenation was improved to match healthy controls [29]. Caranovic et al. reported a study of 102 individuals in which they could stratify healthy volunteers from patients with intermittent claudication (IC) [30]. Using dual wavelength measurements of the triceps surae muscle, they found that baseline sO_2_ in IC patients was relatively hypoxic particularly during a heal-raise exercise. Merdasa et al. observed rapid [31] hypoxiation in the fingers of human volunteers following inflation of a cuff, and found much more severe hypoxiation in the vasculature of the dermis than deeper arteries based on multiwavelength spectral unmixing [31].

Trends in blood perfusion recovery between young and old mice observed by LSCI (Figure 6) were consistent with MSOT (Figure 4). LSCI has been frequently reported for studies of PAD [32], including evaluation of therapeutic interventions [33] and comparison with MSOT [27]. Optical imaging is technically much easier to perform. It reveals skin perfusion, but not depth-resolved tissue heterogeneity. Commercial optical systems for LSCI are typically much cheaper than photoacoustic devices (~$60,000 vs. ~$500,000), but they only show vascular perfusion and not oxygenation. Moreover, light provides poor tissue penetration so that signals report perfusion of the dermis rather than deep muscle or arteries. It is thus significant that our results were consistent between modalities. LSCI has also been applied in human studies to assess diabetic foot neuropathy and peripheral artery disease [34].

It was previously reported that 12-week-old C57BL/6 mice displayed recovery as early as Day 7 following arterial ligation [35,36,37]. Rivard et al. reported faster recovery in blood perfusion in younger animals compared to older ones, using C57BL/6 mice and New Zealand White rabbits [38]. Differences in recovery rates may be attributed to age-related impairments in vascular function and remodeling. Our results indicate that blood perfusion recovery in older mice was reduced compared to young mice following ischemic injury (Figure 6). Blood perfusion recovery in older mice plateaued at 70–80%, consistent with expectations for PAD models [39]. Both BALB/c and C57BL/6 mice are commonly used for hindlimb ischemia models due to their genetic homogeneity [40]. However, BALB/c mice are reported to recover more slowly and exhibit less collateral remodeling, making them more susceptible to necrosis after hindlimb ischemia [36,37,40]. This makes old BALB/c mice particularly suitable for studying chronic PAD, as their response to ischemia is less robust than that of younger mice or other strains.

An increase in adipose in injured tissues with increased cellular density was observed with additional nuclei and other non-muscle cells (e.g., inflammatory cells) present between the fibers. The clustering of nuclei is indicative of an inflammatory response (Figure 7A), as commonly observed in PAD tissues and contributes to further tissue damage and fibrosis. These changes indicate active tissue remodeling after ischemic injury. Notably, young mice exhibited a higher abundance of adipocytes compared to old mice (Figure 7A,B), consistent with reduced adipogenic capacity as reported previously [41,42]. Adipose tissue has been reported to interact with endothelial and inflammatory cells and may influence angiogenesis, inflammatory signaling, and tissue repair through secreted cytokines and growth factors. [43]. Expansion of adipose tissue has been reported in cardiovascular disease and associated with hypoxia [43]. In the present study, however, adipocyte lineage markers, adipokines, and inflammatory cell markers were not measured. Therefore, the increased adipose area should be interpreted as structural evidence of ischemia-associated remodeling, while its specific functional role remains to be clarified in future studies. Overall, these histological changes are consistent with the age-dependent differences in oxygenation and perfusion recovery observed by MSOT and LSCI.

In both young and old mice the gastrocnemius muscle showed significantly elevated collagen in the injured legs compared with the healthy ones (Figure 8A,B), likely due to increased chronic inflammation enhancing fibrotic responses [44]. Moreover, the legs of old mice showed significantly greater fibrosis than the young ones consistent with reported fibroblast senescence in aged tissue increasing fibrogenic potential, extracellular matrix (ECM) production, and overall fibrosis [45]. Alves et al. (2021) found that aging leads to increased iron accumulation in skeletal muscle, which impairs muscle regeneration after ischemia–reperfusion injury [46]. As a result, old mice show poorer muscle repair and increased tissue damage compared with young mice.

Differences in blood perfusion recovery observed using LSCI can be partly explained by age-related alterations in vascular remodeling at the cellular and molecular level. Increased CD31 expression in young mice compared to older mice indicates enhanced angiogenic capacity. While CD31 is widely used as an endothelial marker, it is also expressed by subsets of hematopoietic and inflammatory cells. In ischemic skeletal muscle, immune cell infiltration, particularly macrophages and neutrophils, occurs alongside angiogenesis, and CD31-positive signals may therefore partially reflect inflammatory infiltrates in addition to endothelial structures. This phenomenon has been documented in hindlimb ischemia models, where inflammatory cell recruitment contributes to vascular remodeling and neovascularization [47,48]. The age-related differences in CD31 expression likely reflect a decline in endothelial progenitor cells and vascular remodeling potential in older animals [49,50]. Angiogenesis, triggered by ischemia and reduced oxygen delivery, compensates for femoral artery occlusion by forming new capillaries and improves perfusion by growing new blood vessels with increased diameter to improve blood flow [51]. While we did not examine VEGF/HIF-1 activity, we note previous studies reporting impaired VEGF/HIF-1α signaling, which could contribute to reduced angiogenic remodeling and delayed vascular recovery in aged ischemic muscle. Prior work has shown that aging is associated with reduced VEGF expression, impaired HIF-1 activity, and diminished angiogenic responses after hindlimb ischemia [38,52,53]. Furthermore, aged endothelial cells exhibit reduced proliferation and migration, further limiting angiogenesis [54,55,56].

Ki67, a marker of cell proliferation typically absent in healthy tissues, indicates proliferative activity under pathological conditions [49]. Ki67 staining revealed greater proliferative activity in injured tissues compared to controls, with young PAD mice exhibiting the highest expression (Figure 10). Nuclear clumping observed in PAD tissues further highlights an inflammatory response that contributes to fibrotic progression.

Overall, these data suggest that age significantly affects both angiogenesis and cell proliferation when comparing healthy to diseased tissue. The faster recovery in younger mice can be explained by their more robust angiogenic and proliferative responses, whereas slower angiogenesis and reduced cellular proliferation in older mice contribute to diminished vascularization and impaired tissue repair [50]. Collectively, these results indicate that younger mice exhibit greater vascular density and regenerative potential in PAD, whereas aging impairs both vascular remodeling and cellular proliferation.

As noted by Lotfi et al., the hindlimb ischemia induced by acute ligation, excision or cauterization of the femoral artery causes acute ischemia, which is somewhat different from the chronic onset of PAD in humans [40]. In humans, CLI generally occurs as a result of a chronic process associated with the build-up of atherosclerotic plaque over many years leading to arterial stenosis. A more complex two-stage mouse model to better simulate human PAD has been reported [57], but was beyond the scope of our current work which sought to demonstrate and compare imaging methods for examining muscle pathophysiology. The reduced flow and hypoxiation observed using the femoral ligation model are in line with human disease, and the spontaneous recovery over weeks provides a model that allows testing of interventions designed to promote recovery.

Many methods are available to measure hindlimb perfusion, and they have evolved over the past 100 years in view of the advantages and limitations [58,59]. Calibrated flow probes can assess perfusion, but are invasive, require precise calibration, and lack the spatial resolution to identify localized ischemic regions, making them less suitable for detailed studies [40]. Microspheres and contrast-enhanced X-ray imaging offer deep tissue sensitivity, but their invasive nature, risk of ionizing radiation, and technical complexity limit practicality. MRI provides high spatial resolution, but is expensive, time-consuming, and often requires contrast agent injection [40]. Nonetheless, it is particularly versatile being able to show both anatomy and specific aspects of pathophysiology such as blood flow, tissues perfusion, oxygenation, pH and necrosis. Contrast-enhanced ultrasound sonography is widely used and can be enhanced in power- or color-Doppler modes or using microbubbles. Here, we examined LSCI and MSOT on separate cohorts of mice and demonstrated broad equivalency of observations. We recognize that it would have been more powerful to undertake both imaging procedures on individual mice, but this was logistically not feasible. It would have also led to longer studies at each time point, causing additional stress for mice to be transferred from one instrument to another.

Multispectral optoacoustic tomography (MSOT) offers high-resolution imaging up to several centimeters deep, enabling visualization of vascular oxygen saturation throughout leg muscles. It is particularly rapid in providing tomographic or volumetric images revealing tissue heterogeneity. We were concerned that hemorrhage, from surgery, or sutures might interfere with imaging, but no obvious problems were encountered. We used a system with 256 transducer elements and note that resolution and contrast typically improve with number of transducers [60], so that the more sophisticated system available with 512 transducers should provide enhanced sensitivity. Additional insights into pathophysiology, receptor expression and therapy can be achieved with contrast agents optimized for PAI [61,62]. For human applications, innovations and technical developments promise further opportunities in imaging PAD and therapeutic interventions. Recent instruments can achieve volumetric images with a single shot providing sO_2_ dynamics in hands and feet with respect to vascular occlusion [63]. Moreover, devices dedicated to specific disease sites, e.g., forearm, can improve sensitivity, patient comfort and utility [16]. An all-optical Fabry–Perot ultrasound sensor has been shown to provide highly detailed 3D microvascular image in a few seconds, or less, by parallelizing the optical architecture of the sensor readout, using excitation lasers with high pulse-repetition frequencies and by exploiting compressed sensing [64]. Developments in artificial intelligence processing of imaging and interpretation may enhance sensitivity and specificity [65].

## 5. Conclusions

This study demonstrates the utility of multispectral optoacoustic tomography (MSOT) and laser speckle contrast imaging (LSCI) as effective, noninvasive tools for assessing vascular oxygenation and blood perfusion in a mouse model of peripheral artery disease (PAD). Our findings reveal pronounced age-dependent differences in vascular recovery, with young mice exhibiting faster and more robust perfusion restoration, angiogenesis, and tissue remodeling compared to older mice. MSOT provided high-resolution insights into dynamic oxygenation patterns, while LSCI confirmed functional trends in blood flow, both of which were supported by histological evidence showing significant reductions in fibrosis and increased proliferative and angiogenic activity in young mice. These results highlight that older mice experience impaired regenerative capacity and heightened chronic inflammation, contributing to slower vascular recovery. Compared to conventional imaging approaches, MSOT offers superior spatial resolution and depth penetration, making it particularly valuable for investigating deep tissue oxygenation in PAD. Collectively, these findings emphasize the critical impact of aging on vascular recovery and validate MSOT and LSCI as powerful modalities for time-course evaluation and therapeutic assessment in PAD research.

## Data Availability

The raw data supporting the conclusions of this article will be made available by the authors upon request.

## Supplementary Materials

The following supporting information can be downloaded at https://www.mdpi.com/article/10.3390/diagnostics16121783/s1, Figure S1: Vascular oxygen saturation (mean sO_2_^MSOT^) in muscles of injured legs of individual young and old mice over 2 weeks following femoral artery cauterization; Figure S2: Vascular oxygen saturation (mean sO_2_^MSOT^) in muscles of injured legs of individual mice with respect to oxygen gas-breathing challenge; Figure S3: Sequential laser speckle contrast imaging (LSCI) of blood perfusion in individual mice.
