# Supplementary material for: Investigating Age-Dependent Oxygenation and Blood Perfusion in a Mouse Model of Peripheral Artery Disease (PAD) Using Multispectral Optoacoustic Tomography (MSOT), Laser Speckle Contrast Imaging (LSCI) and Histology
Source: Diagnostics (Basel). 2026 Jun 9;16(12):1783. doi: 10.3390/diagnostics16121783 (PMC13297732; doi:10.3390/diagnostics16121783)
Supplement: Supplementary file 1 [file diagnostics-16-01783-s001.zip › diagnostics-4254562-supplementary.pdf]

Investigating Age-Dependent Oxygenation and Blood Perfusion in a Mouse Model of Peripheral Artery Disease (PAD) using Multispectral Optoacoustic Tomography (MSOT), Laser Speckle Contrast Imaging (LSCI) and Histology

Bushra Afzal, Vy Tran, Na Nguyen, Savannah Qui-Tam Le, Tam Nguyen, Kytai T. Nguyen, Li Liu and Ralph P. Mason

Supplementary Figures

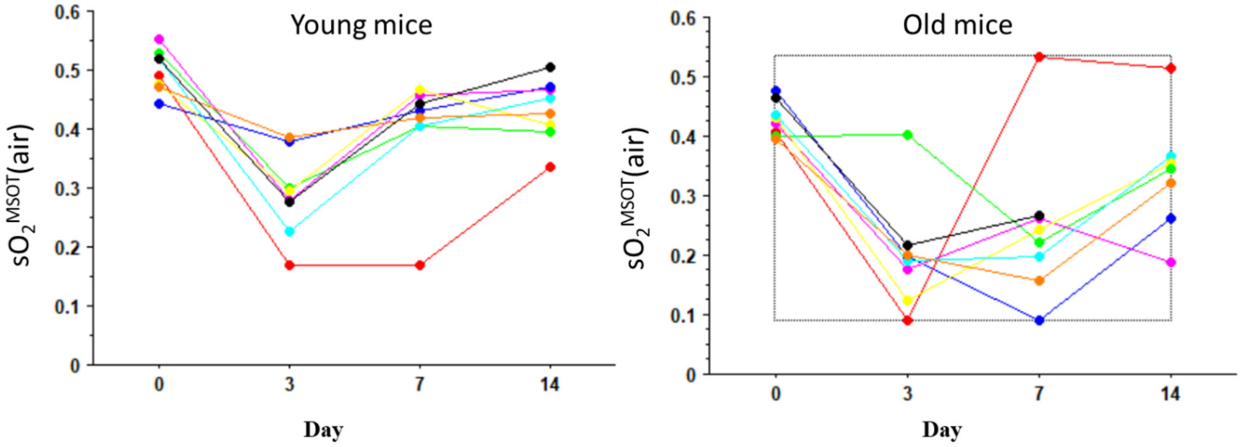

Supplementary Figure S1. Vascular oxygen saturation (mean sO_2^{MSOT}) in muscles of injured legs of individual young and old mice over 2 weeks following femoral artery cauterization.

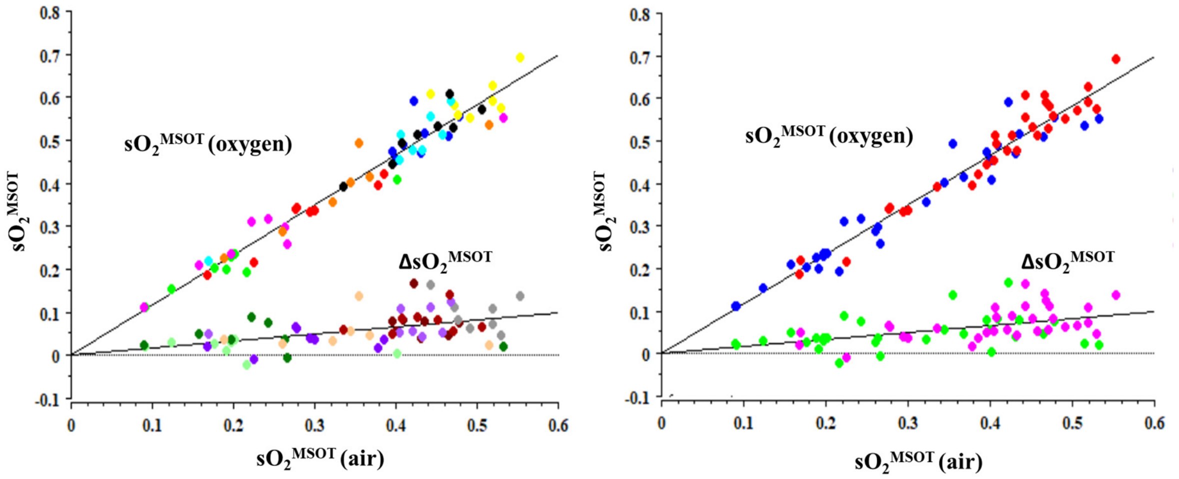

Supplementary Figure S2. Vascular oxygen saturation (mean sO_2^{MSOT}) in muscles of injured legs of individual mice with respect to oxygen gas-breathing challenge. **Left)** Data shown for repeat measurements over 2 weeks (pre- and post-surgery) for all individual mice (young and old) identified by separate colors comparing mean sO_2^{MSOT}, while breathing oxygen to baseline air and showing the response to the oxygen gas breathing challenge (mean ΔsO_2^{MSOT}). D0 (dark blue and yellow), D3 (red and light blue), D7 (green and orange), D14 (pink and black). **Right)** Same data but discriminated based on age of mice: red and purple (young mice), blue and green (old mice). sO_2^{MSOT} depended strongly on baseline (R^2>0.94), while the difference showed much weaker correlation (R^2=0.251).

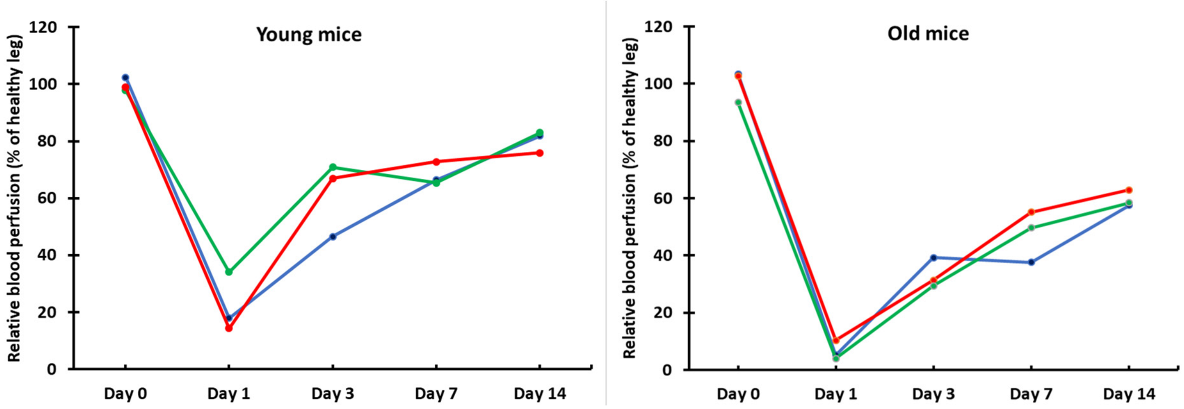

Supplementary Figure S3. Sequential laser speckle contrast imaging (LSCI) of blood perfusion in individual mice. **Left)** Comparison of sequential relative blood perfusion in injured paw vs. control over 2 weeks following surgery (D0, D1, D3, D7 and D14) in 3 individual young mice. **Right)** Equivalent data in 3 individual old mice. In each case the mice behaved quite consistently.
